# Health information technology interventions and engagement in HIV care and achievement of viral suppression in publicly funded settings in the US: A cost-effectiveness analysis

**DOI:** 10.1371/journal.pmed.1003389

**Published:** 2021-04-07

**Authors:** Starley B. Shade, Elliot Marseille, Valerie Kirby, Deepalika Chakravarty, Wayne T. Steward, Kimberly K. Koester, Adan Cajina, Janet J. Myers

**Affiliations:** 1 Institute for Global Health Sciences, Department of Epidemiology and Biostatistics, University of California, San Francisco, California, United States of America; 2 Center for AIDS Prevention Studies, University of California, San Francisco, California, United States of America; 3 Demonstration and Evaluation Branch, HIV/AIDS Bureau, Health Resources and Services Administration, Rockville, Maryland, United States of America; University of Washington, UNITED STATES

## Abstract

**Background:**

The US National HIV/AIDS Strategy (NHAS) emphasizes the use of technology to facilitate coordination of comprehensive care for people with HIV. We examined cost-effectiveness from the health system perspective of 6 health information technology (HIT) interventions implemented during 2008 to 2012 in a Ryan White HIV/AIDS Program (RWHAP) Special Projects of National Significance (SPNS) Program demonstration project.

**Methods/findings:**

HIT interventions were implemented at 6 sites: Bronx, New York; Durham, North Carolina; Long Beach, California; New Orleans, Louisiana; New York, New York (2 sites); and Paterson, New Jersey. These interventions included: (1) use of HIV surveillance data to identify out-of-care individuals; (2) extension of access to electronic health records (EHRs) to support service providers; (3) use of electronic laboratory ordering and prescribing; and (4) development of a patient portal. We employed standard microcosting techniques to estimate costs (in 2018 US dollars) associated with intervention implementation. Data from a sample of electronic patient records from each demonstration site were analyzed to compare prescription of antiretroviral therapy (ART), CD4 cell counts, and suppression of viral load, before and after implementation of interventions. Markov models were used to estimate additional healthcare costs and quality-adjusted life-years saved as a result of each intervention. Overall, demonstration site interventions cost $3,913,313 (range = $287,682 to $998,201) among 3,110 individuals (range = 258 to 1,181) over 3 years. Changes in the proportion of patients prescribed ART ranged from a decrease from 87.0% to 72.7% at Site 4 to an increase from 74.6% to 94.2% at Site 6; changes in the proportion of patients with 0 to 200 CD4 cells/mm^3^ ranged from a decrease from 20.2% to 11.0% in Site 6 to an increase from 16.7% to 30.2% in Site 2; and changes in the proportion of patients with undetectable viral load ranged from a decrease from 84.6% to 46.0% in Site 1 to an increase from 67.0% to 69.9% in Site 5. Four of the 6 interventions—including use of HIV surveillance data to identify out-of-care individuals, use of electronic laboratory ordering and prescribing, and development of a patient portal—were not only cost-effective but also cost saving ($6.87 to $14.91 saved per dollar invested). In contrast, the 2 interventions that extended access to EHRs to support service providers were not effective and, therefore, not cost-effective. Most interventions remained either cost-saving or not cost-effective under all sensitivity analysis scenarios. The intervention that used HIV surveillance data to identify out-of-care individuals was no longer cost-saving when the effect of HIV on an individual’s health status was reduced and when the natural progression of HIV was increased. The results of this study are limited in that we did not have contemporaneous controls for each intervention; thus, we are only able to assess sites against themselves at baseline and not against standard of care during the same time period.

**Conclusions:**

These results provide additional support for the use of HIT as a tool to enhance rapid and effective treatment of HIV to achieve sustained viral suppression. HIT has the potential to increase utilization of services, improve health outcomes, and reduce subsequent transmission of HIV.

## Abstract 2

Starley Shade and co-workers assess cost-effectiveness of information technology interventions in HIV care programs in the United States.

## Author summary

**Why was this study done?:**

- The Health Resources and Services Administration’s Special Projects of National Significance Program (HRSA/SPNS) funded a 4-year initiative (2007 to 2011) in 6 demonstration sites to enhance and evaluate existing health information electronic network systems for people living with HIV (PLHIV) in underserved communities.
- Each of the 6 demonstration sites implemented one or more health information technology (HIT) interventions to facilitate comprehensive care and enhance engagement in HIV medical services. These interventions included: (1) use of HIV surveillance data to identify out-of-care individuals; (2) extension of access to electronic health records to support service providers; (3) use of electronic laboratory ordering and prescribing; and (4) development of a patient portal.
- This study estimates the total costs, cost-effectiveness, and potential cost-savings of these 6 interventions.

**What did researchers do and find?:**

- We used information on the cost of each intervention and the health status of PLHIV in each setting before and after implementation of each intervention to estimate: (1) changes in the cost of care and other services for PLHIV in each setting; and (2) changes in expected health status (measured as quality-adjusted life-years or QALYs) among PLHIV in each setting. We then used this information to estimate additional healthcare costs and QALYs gained for each intervention.
- Four of the interventions were associated with lower healthcare costs and better health outcomes (QALYs gained) for PLHIV in each setting. These interventions saved between $6.87 and $14.91 per dollar invested.
- Two interventions that provided access to medical record information to support service providers were not associated with improved health outcomes for PLHIV in these settings. These interventions were not effective or cost-effective.

**What do these findings mean?:**

- These results show that HIT interventions that facilitate changes in patient or provider behavior have the potential to improve the health status of PLHIV and reduce healthcare costs. HIT interventions that only provided additional information to support service providers were less successful.
- This study did not include a contemporaneous comparison group. Therefore, we do not know the degree to which improvements in the health status of PLHIV in these settings were due to changes in the quality of care for PLHIV over the life of the interventions.

## Introduction

The United States National HIV/AIDS Strategy (NHAS), first introduced in 2010 and subsequently revised in 2015, has as its goals: to reduce the number of people who become infected with HIV; increase access to care and optimize health outcomes for people living with HIV (PLHIV); reduce HIV-related health disparities; and achieve a more coordinated national response to the HIV epidemic [1]. The strategy articulates measurable action steps and sets 5-year quantitative targets based on evidence-based approaches. Its stated purpose is to provide a roadmap for responding to the epidemic among public and private stakeholders; its guiding principles include accountability and science-driven decision-making.

The NHAS targets to increase the proportion of all HIV–infected individuals in the US who are aware of their serostatus, linked to care within 3 months of diagnosis, engaged in HIV care continuously and ultimately achieve suppression of the virus in their blood [2,3]. Achieving these targets would contribute to ending the epidemic because suppression of viral load is important for individual patient health and for the prevention of transmission of HIV in the population as a whole [4].

Unfortunately, there are numerous barriers to “rapid and effective" HIV care [5–12], including structural challenges (e.g., lack of housing), insufficient financing (e.g., lack of insurance) [13,14], personal and cultural characteristics (e.g., beliefs about health system, racism), comorbidities (e.g., mental illness and substance abuse), stigma, fear of confidentiality violations, and healthcare provider attitudes [5–24]. Research suggests that both individual and system-level interventions have the potential to improve care engagement and the quality of HIV medical and support services [3,25].

To date, few studies have explored the extent to which sharing patient information across geographically disparate HIV surveillance, primary care, and support service organizations can enhance linkage to care, retention and adherence to care and treatment, the quality of core and support services, as well as health outcomes for people with HIV. This sharing process, however, is key to achieving rapid and effective HIV care precisely because of the multifaceted impacts on care outcomes listed above. For example, the National HIV/AIDS Strategy: Updated to 2020 (NHAS 2020) goals call for enhanced collaboration among providers, improved assessments and measurements of health outcomes to track progress towards NHAS 2020 goals across populations in a region [26]. Effectively meeting these objectives requires technologies and practices that ensure that providers and the communities in which they are embedded coordinate the services that they deliver to patients.

Health information technology (HIT) interventions, including the electronic transfer of information between organizations (health information exchange), extending access to information with additional providers within organizations, and sharing of information with patients has the potential to enhance engagement in comprehensive HIV care. HIT interventions have been previously implemented in the context of other diseases to link public health surveillance programs to primary care services, laboratory and pharmacies to primary care, and primary and specialty care [27–31]. HIT has the potential to close many of the gaps that lead to suboptimal care for PLHIV by, for example, improving the referral and tracking of patients among services, identifying patients who have missed appointments, and allowing providers to coordinate services to ensure that each individual receives a comprehensive set of services that they need.

To test the potential of HITs in supporting better-coordinated care in publicly funded settings, the Health Resources and Services Administration (HRSA) sponsored an initiative to develop HIT interventions in clinical care sites serving patients eligible for the Ryan White HIV/AIDS Program (RWHAP). The RWHAP was enacted by Congress in 1990 to ensure access to quality HIV care and treatment for those who cannot afford it, provision of support services (e.g., transportation and housing) for those who experience challenges and/or obstacles in entering and remaining in care, and coordination of care (e.g., medical case management) for those who have co-occurring conditions that impact the effectiveness of HIV care [32,33]. RWHAP providers typically deliver a constellation of services to support HIV status awareness, linkage to care, and retention. Each of the demonstration sites in the initiative implemented systems that supported engagement in care by targeting gaps they had identified in their clinical settings.

## Methods

### Description of the initiative

One component of the RWHAP is the Special Projects of National Significance (SPNS) Program, which provides grants to fund innovative models of care and supports the development of effective and innovative delivery systems for HIV care. In 2007, SPNS funded a 4-year initiative in 6 demonstration sites to enhance and evaluate existing health information electronic network systems for PLHIV in underserved communities. These 6 demonstration sites were: the Bronx-Lebanon Hospital Center in Bronx, New York; the City of Paterson Department of Human Services, New Jersey; the Duke University Center for Health Policy in Durham, North Carolina; the Louisiana State University Health Services Center in New Orleans, Louisiana; the New York Presbyterian Hospital in New York, New York; and the St. Mary Medical Center Foundation in Long Beach, California. The University of California, San Francisco (UCSF) Center for AIDS Prevention Studies was funded as the Evaluation and Support Center (hereafter referred to as the “Center”) to conduct a cross-site evaluation of the HIT interventions and provide technical assistance and support to the demonstration sites. This paper estimates the total costs, cost-effectiveness, and potential cost-savings of each intervention in the initiative according to the CHEERS Statement (see S1 CHEERS Checklist).

### Ethics statement

The protocol for the cross-site evaluation was approved by the Committee for Human Research at UCSF (see S1 Evaluation Protocol). However, this protocol does not include information on how information about the cost-effectiveness of demonstration site interventions will be evaluated as our cost-effectiveness analysis does not include any identifiable data as thus is deemed exempt from UCSF IRB approval.

### HIT interventions

Each of the 6 demonstration sites implemented one or more HIT interventions to facilitate comprehensive care and enhance engagement in HIV medical services. These interventions and the characteristics of patients within each demonstration site have been previously detailed [34–37] and are summarized here:

- Site 1 developed a structured patient summary within its electronic health record (EHR) that included highlighted alerts to identify needed clinical services. The hospital also provided case managers with access to these patient summaries to facilitate coordination of support services and reinforce engagement in HIV care. This site targeted “high need patients,” defined as patients who had detectable viral loads despite multiple medical and social support service visits across multiple social service providers. This site anticipated that providing access to medical record information to cases managers would facilitate coordination of care and reduction of redundancies in the provision of support services.
- Site 2 provided support service providers access to a regional medical center EHR to facilitate coordination of support services and reinforce engagement in HIV care. This site anticipated that improved coordination of care would result in higher utilization of necessary support services.
- Site 3 linked the state HIV surveillance branch and EHRs in publicly funded health facilities. It created an alert whenever a patient known to the surveillance branch to be out of care for HIV treatment presented for services in an emergency room or other non-HIV healthcare setting. Providers at the care site then acted on the alert to facilitate reengaging the patient in HIV care. This site anticipated that the intervention would improve linkage or re-engagement in care among previously out-of-care individuals.
- Site 4 created continuity of care patient summaries. Patients were then given to access this information through a patient portal to facilitate engagement in HIV care. Patients were also able to provide this information to external providers to facilitate coordination of services. This site anticipated that access to information would increase utilization of necessary care and support services.
- Site 5 developed an EHR with summary comparison reports that was shared across all health service providers. This shared record facilitated the development of a quality improvement framework in which activities were implemented to increase targeted prevention services. Health record alerts were also formulated to help providers identify and intervene with patients who had not received needed clinical services. This site anticipated that the intervention would improve the quality of care for HIV–infected patients.
- Site 6 implemented HIT to facilitate electronic prescriptions as well as laboratory work orders to reduce the time needed to access these services and to enhance engagement in HIV care.

### Data collection

The Center collected de-identified quantitative electronic patient record data from each demonstration site for the 6-month period preceding the implementation of the HIT intervention and for each 6-month period thereafter through the end of the project. Demonstration sites were asked to provide a simple random sample of at least 100 patients from each demonstration site; however, some sites chose to provide records for all patients at each time point and some sites chose to submit information for a larger random sample of patients. The present analyses include data for the 6 months prior to the intervention implementation and after full implementation of the intervention (during the last reporting period).

### Measures

Data elements included as outcomes or effectiveness measures in the present analysis include:

- Prescription of antiretroviral therapy (ART): A binary outcome variable was created to record if the client had been prescribed highly active ART at any time during a reporting period.
- CD4 cell count: A categorical variable was created to record if the client had a low (0 to 200 cells/mm^3^), medium (201 to 500 cells/mm^3^), or high (>500 cell/mm^3^) CD4 cell count.
- Undetectable viral load: For this evaluation, undetectable viral load was defined as less than 1,000 copies/mL because this was the level of detection that was available to the site using data from the HIV surveillance system. A binary outcome variable was created to record if the client had an undetectable viral load at the last test during a reporting period.

### Measuring intervention costs

We used standard micro-costing techniques to estimate the incremental cost (in 2018 US dollars) of implementation of each site’s intervention from the perspective of the health system [38]. We developed a standard cost data collection tool and protocol. Demonstration site staff completed the data collection tool annually from September 2008 through August 2010. We included additional personnel time, materials, and other resources needed to implement the interventions, whether funded through the SPNS initiative (direct costs) or through other sources (in-kind costs). Sites were granted the same amount annually. Sites used some of these resources to conduct evaluation of their local interventions. These costs were not included in estimation of intervention costs. Costs were adjusted for inflation to 2018 US dollars (https://data.bls.gov/cgi-bin/cpicalc.pl).

Direct and in-kind expenditures were classified in 1 of 4 categories: (i) personnel, including fringe benefits; (ii) other recurring costs consisting of supplies and services; (iii) capital expenditures; and (iv) building space. Capital expenditures such as computers and furniture were amortized over 5 years of expected useful life and assuming no salvage value. Building space was valued at the market rental rate for any space that had previously been utilized for another activity.

In order to understand the activities and associated costs needed to implement each intervention, we worked with demonstration site staff to develop a comprehensive list of personnel activities. These included: HIT design and customization; initial debug-quality control; system monitoring and quality control; ongoing use of system; ongoing maintenance including data backup; system refinement and improvement; user support services; stakeholder sessions; patient recruitment; training of providers; training of case managers; training of patients; and legal and other activities. Demonstration site staff distributed staff time across these 14 activities on a monthly basis and then totaled for the year and multiplied by the compensation rate. The allocation was performed based on the staff member’s knowledge of intervention operations and their understanding of how personnel spend their time between HIT and non-HIT activities. Consultant and other outside costs were entered as direct dollar amounts assigned to each activity such that the total equaled the sum spent for each outside contracted service.

### Measuring effectiveness

Program-attributable health outcomes were obtained by estimating quality-adjusted life-years (QALYs) gained among patients impacted by the interventions, using information on prescription of ART, CD4 cell count and viral suppression at baseline and at last follow-up. We calculated expected QALYs over a 5-year period based on the distribution of patient health states at baseline and follow-up using a Markov health state transition model, literature-derived estimates of transition probabilities and health state utilities, and a per annum discount of 3% (see Tables A-E in S1 Text) [39]. We then compared expected QALYs at baseline and follow-up to estimate QALYs gained.

### Measuring healthcare costs

We estimated the cost of healthcare with and without implementation of the interventions by applying literature-derived costs of healthcare (adjusted for inflation to 2018 US dollars) to the distribution of patients by CD4 cell count, viral suppression, and ART before and after implementation of each intervention (see Tables A and E in S1 Text) [41,47–50]. We then estimated the total 5-year cost of healthcare with and without the interventions based on the number of patients exposed to each intervention. For each demonstration site, our outcomes were obtained by taking the difference in costs with and without implementation of the intervention.

### Assessing cost-effectiveness

We estimated the cost-effectiveness of each intervention using pre-post analysis. We added the difference in modelled 5-year costs of healthcare with and without HIT implementation to the cost of each intervention. This number was then divided by the estimated number of QALYs gained among patients exposed to the interventions to estimate the cost per QALY gained for each intervention. If the difference in 5-year costs of healthcare (with minus without) was greater than the cost of the intervention, then the intervention was considered to be “cost saving.” In this case, cost savings were estimated as the reduction in healthcare costs over the cost of the intervention.

### Sensitivity analysis

We estimated the percent change in cost per QALY gained when we varied ART and healthcare costs (based on previous literature; see Table E in S1 Text), utilities (200%, 50%) and transition probabilities (200%, 50%) for each intervention in order to examine the consistency of our observed results across participating sites.

## Results

Overall, demonstration site interventions cost $3,913,313 over 3 years (range = $287,682 to $998,201; Table 1). This included $3,648,512 in direct costs (range = $224,968 to $889,409) in funding from the HRSA SPNS initiative and $412,289 in indirect (in-kind) costs (range = $24,227 to $199,386) with external funding from demonstration sites and their partners. Personnel effort made up the majority of costs across all intervention sites (overall = 83%; range = 74% to 91%) with a smaller proportion going to recurring goods and services and facility rental (overall = 14%; range = 6% to 26%). Capital equipment (including computers and informatics infrastructure) comprised only a small percentage of costs across all demonstration sites (overall = 3%; range = 1% to 6%).

**Table 1:** Costs of HIT interventions by resources category and site.

Cost category	Site 1	Site 2	Site 3	Site 4	Site 5	Site 6	Total
**Direct Costs**							
Personnel	$765,603 (92%)	$147,664 (66%)	$500,093 (83%)	$610,774 (69%)	$675,570 (91%)	$293,314 (81%)	$2,993,018 (82%)
Recurring Costs	$36,820 (4%)	$63,633 (28%)	$84,818 (14%)	$246,168 (28%)	$54,570 (7%)	$47,850 (13%)	$533,859 (15%)
Capital Costs*	$25,582 (3%)	$13,671 (6%)	$17,456 (3%)	$32,467 (4%)	$10,298 (1%)	$22,161 (6%)	$121,635 (3%)
Total	**$828,005**	**$224,968**	**$602,367**	**$889,409**	**$740,438**	**$363,325**	**$3,648,512**
**In-kind Costs**							
Personnel	$13,139 (54%)	$62,714 (100%)	$45,813 (100%)	$124,992 (63%)	$11,011 (69%)	$33,856 (53%)	$291,525 (71%)
Recurring Costs	$11,008 (46%)	$0 (0%)	$0 (0%)	$73,338 (37%)	$4,965 (31%)	$30,245 (47%)	$119,636 (29%)
Capital Costs*	$0 (0%)	$0 (0%)	$0 (0%)	$1,056 (1%)	$72 (0%)	$0 (0%)	$1,128 (0%)
Total	**$24,227**	**$62,714**	**$45,813**	**$199,386**	**$16,048**	**$64,101**	**$412,289**
**Total Costs**							
Personnel	$778,742 (91%)	$210,378 (73%)	$545,906 (84%)	$735,766 (74%)	$686,581 (91%)	$327,170 (77%)	$3,237,649 (83%)
Recurring Costs	$47,908 (6%)	$63,633 (22%)	$84,818 (13%)	$218,912 (22%)	$59,535 (8%)	$78,095 (18%)	$552,901 (14%)
Capital Costs*	$25,582 (3%)	$13,671 (5%)	$17,456 (3%)	$33,523 (3%)	$10,370 (1%)	$22,161 (5%)	$122,763 (3%)
Total	**$852,232**	**$287,6828**	**$648,180**	**$988,201**	**$756,486**	**$427,426**	**$3,913,313**

*Costs amortized over the life of the equipment.

Table 2 includes the distribution of personnel costs by activity across demonstration sites. Overall, costs associated with personnel effort were evenly divided across conceptualization and design of the interventions (overall = 30%; range = 10% to 55%), ongoing system maintenance and improvement (overall = 30%; range = 13% to 39%), and user training and support (overall = 32%; range = 8% to 55%, although the distribution across these 3 broad categories varied substantially across interventions. Three (3) demonstration sites also incurred legal costs associated with individual and system-level privacy issues (overall = 8%; range = 0% to 19%).

**Table 2:** Distribution of personnel costs by activity and site (costs in thousands).

Activity	Site 1 $ (%)	Site 2 $ (%)	Site 3 $ (%)	Site 4 $ (%)	Site 5 $ (%)	Site 6 $ (%)	Total $ (%)
**Initial Design and Programming**	**$276** **(35%)**	**$24** **(11%)**	**$126** **(23%)**	**$77** **(10%)**	**$376** **(55%)**	**$114** **(35%)**	**$987** **(30%)**
ENS design and customization	$248	$17	$91	$58	$235	$86	$731
Initial debug-quality control	$28	$7	$35	$19	$141	$28	$256
**Ongoing maintenance and Improvement**	**$101** **(13%)**	**$80** **(38%)**	**$214** **(39%)**	**$237** **(32%)**	**$253** **(37%)**	**$96** **(29%)**	**$964** **(30%)**
System monitoring; quality control	$27	$9	$36	$65	$173	$18	$325
Ongoing use of system	$24	$57	$58	$0	$27	$9	$163
Ongoing maintenance; data back-up	$3	$3	$61	$155	$33	$29	$285
System refinement, improvement	$47	$811	$59	$18	$20	$40	$192
**User training and support**	**$253** **(32%)**	**$104** **(50%)**	**$151** **(28%)**	**$402** **(55%)**	**$58** **(8%)**	**$82** **(25%)**	**$1,026** **(32%)**
User support services	$24	$14	$22	$45	$35	$33	$170
Stakeholder sessions	$65	$35	$116	$104	$0	$38	$351
Patient recruitment into ENS	$65	$13	$0	$85	$5	$0	$165
Training of providers	$34	$2	$10	$22	$0	$9	$77
Training of case managers	$31	$41	$2	$61	$0	$1	$127
Training of patients	$33	$0	$0	$85	$17	$0	$136
**Other**	**$150** **(19%)**	**$2** **(1%)**	**$54** **(10%)**	**$20** **(3%)**	**$0** **(0%)**	**$35** **(11%)**	**$261** **(8%)**
Legal	$38	$2	$54	$0	$0	$33	$128
Other	$111	$0	$0	$20	$0	$2	$133
**Total**	**$779**	**$210**	**$546**	**$736**	**$687**	**$327**	**$3,238**

Table 3 includes the distribution of prescription of ART, CD4 cell count, and viral load suppression within sites at baseline and after full HIT intervention implementation. We observed increases in the proportion of patients on ART in Sites 2 (from 81.1 to 90.5), 3 (from 54.0 to 59.0), 5 (from 85.5 to 87.7), and 6 (from 74.6 to 94.2). In contrast, we observed decreases in the proportion on patients on ART in Sites 1 (from 91.0 to 82.9) and 4 (from 87.0 to 72.9). We observed small differences in the distribution of CD4 cell counts within demonstration sites. Of note, the proportion of patients with 0 to 200 CD4 cells/mm^3^ increased in Sites 1 (from 16.7 to 30.2) and 2 (from 14.8 to 17.8), which may have been indicative of the targeting of out-of-care clients that occurred in these sites. In contrast, we observed decreases in the proportion of patients with 0 to 200 CD4 cells/mm^3^ in Sites 3 (31.8 to 29.4), 4 (17.4 to 15.2), 5 (20.2 to 11.0), and 6. We observed small changes in the proportion of patients with undetectable viral load in Sites 2 (62.2 to 63.5), 5 (67.0 to 69.9), and 6 (72.2 to 68.0) and large decreases in the proportion of patients with undetectable viral load in Sites 1 (84.6 to 46.0), 3 (34.8 to 9.0), and 4 (39.3 to 24.2).

**Table 3:** Patient ART status, CD4 cell count, and viral suppression at baseline and follow-up.

	Site 1		Site 2		Site 3		Site 4		Site 5		Site 6	
	BL	FU	BL	FU	BL	FU	BL	FU	BL	FU	BL	FU
	*n = 78%*	*n = 374%*	*n = 196%*	*n = 200%*	*n = 100%*	*n = 100%*	*n = 100%*	*n = 99%*	*n = 117%*	*n = 219%*	*n = 500%*	*n = 500%*
**% on ART**	91.0	82.9	81.1	90.5	54.0	59.0	87.0	72.7	85.5	87.7	74.6	94.2
**CD4 at last test**												
**0–200**	16.7	30.2	14.8	17.8	31.8	29.4	17.4	15.2	20.2	11.0	12.2	8.4
**201–500**	47.2	40.5	49.5	36.7	43.2	47.1	39.1	34.8	42.2	33.5	47.1	38.3
**>500**	36.1	29.3	35.7	45.6	25.0	23.5	43.5	50.0	37.6	55.5	40.7	53.3
**% with undetectable VL**	84.6	46.0	62.2	63.5	34.8	9.0	39.3	24.2	67.0	69.9	72.2	68.0

ART, antiretroviral therapy; BL, baseline; FU, follow-up; VL, viral load.

Table 4 includes information on predicted healthcare costs and QALYs within the exposed population based on prescription of ART, CD4 cell counts, and viral suppression before and after HIT intervention implementation in each site. This information was combined with information about the cost of each intervention to estimate cost-effectiveness and cost savings. Prior to HIT intervention implementation, predicted healthcare costs were high (range = $82,250 to $100,067 per patient per year) and predicted QALYs were low (range = 3.71 to 4.12 per exposed patient) across sites at baseline. We observed a decline in predicted healthcare and intervention costs (range = $68,397 to $94,392 per patient per year; change in costs per patient: Site 1 = $29,244; Site 2 = $8,835; Site 3 = −$15,869; Site 4 = −$20,852; Site 5 = −$36,196; Site 6 = −$4,695) and small changes in predicted QALYs (range = 3.68 to 4.18 per patient; change in additional QALYs per patient: Site 1 = −0.44; Site 2 = −0.01; Site 3 = 0.01; Site 4 = 0.03; Site 5 = 0.08; Site 6 = 0.02) after HIT intervention implementation due to changes in prescription of ART and consequent improvements in CD4 cell counts and viral suppression among patients in the intervention sites.

**Table 4:** Cost-effectiveness and return on investment for HIT interventions.

	Site 1	Site 2	Site 3	Site 4	Site 5	Site 6
**Exposed**						
	350	649	258	409	263	1,181
**Costs before intervention implementation (costs in thousands)**						
Health care costs (5 years)	$162,754	$289,621	$129,087	$186,690	$122,785	$515,211
QALYs	1443.29	2625.31	958.20	1598.89	1053.02	4863.91
$/QALY	$113	$110	$135	$117	$117	$106
**Cost after intervention implementation (costs in thousands)**						
Health care costs (5 years)	$177,333	$297,869	$122,017	$172,549	$111,505	$507,164
Intervention costs (3 years)	$852	$288	$648	$1,089	$756	$427
Total costs	$178,185	$298,157	$122,655	$173,637	$112,261	$507,592
QALYs	1288.01	2618.10	959.64	1709.97	1073.22	4932.77
$/QALY	$138	$114	$128	$101	$105	$103
**Cost-effectiveness (costs in thousands)**						
Additional costs	$15,432	$8,536	$(4,458)	$(14,141)	$(10,524)	$(7,620)
Additional cost per person	$44	$13	$(17)	$(35)	$(40)	$(6)
Additional QALYs	−155.28	−7.22	1.44	13.99	20.20	18.83
$/QALY	Dominated	Dominated	Cost Saving	Cost Saving	Cost Saving	Cost Saving
**Cost savings (per $1 invested)**						
	None	None	$6.87	$13.99	$14.91	$12.97

HIT, health information technology; QALY, quality-adjusted life-year.

In 4 sites (Sites 3 to 6), the decline in predicted healthcare costs more than offset the cost of the HIT interventions suggesting that these interventions could be not only cost-effective but also cost saving. In these sites, each dollar invested was associated with a predicted decline in healthcare costs of $6.32 to $12.97. In 2 sites, (Sites 1 and 2), we observed a decline in predicted QALYs among patients exposed to their interventions. Thus, these results suggest that these interventions were not effective and, thus, not cost-effective.

**Fig 1:**
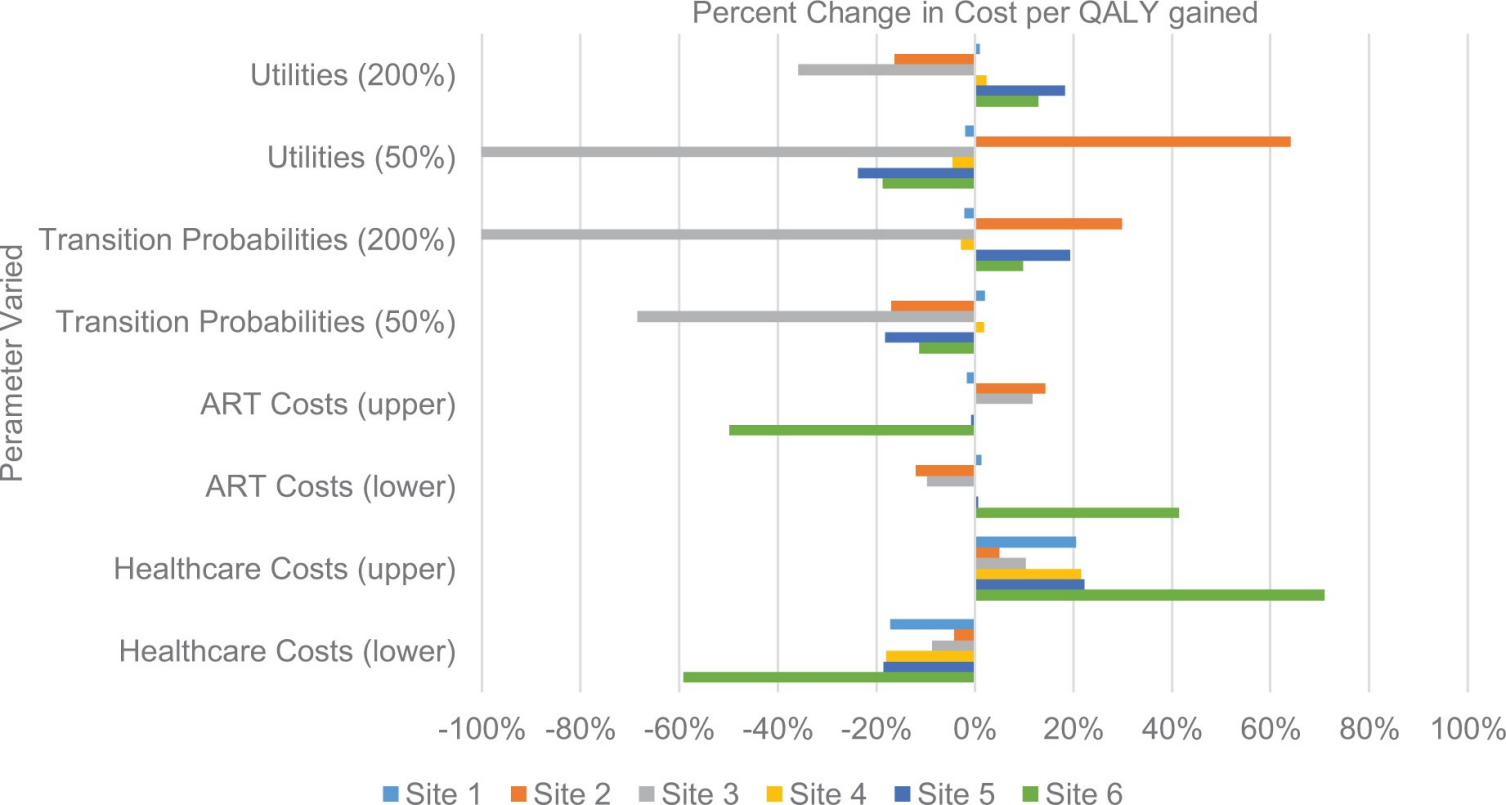
Sensitivity analysis.

Fig 1 includes results of univariate sensitivity analysis conducted by varying ART costs, healthcare costs, utilities, and transition probabilities. We estimated the percent change in cost per QALY gained under various scenarios. Site 6, which served the largest number of patients, was sensitive to variation in ART and healthcare costs, while Sites 2 and 3, which produced fewer additional QALYs, were sensitive to variation in utilities and transition probabilities. Cost per QALY gained varied less than 20% under all scenarios for Sites 1, 4, and 5. Of note, the status of each intervention as not effective or cost saving was consistent across all almost all scenarios. Site 3 was no longer cost saving when the effect of HIV on a patient’s health was cut in half (utility 50%; cost per QALY gained = $39,397,160) and when the probability of transition from one disease state to another was doubled (transition probability 200%; cost per QALY gained = $3,087,353).

## Discussion

To our knowledge, this initiative is the first to demonstrate that HIT may not only improve engagement in HIV care and clinical outcomes but may also reduce costs associated with HIV care and treatment. Previous research has shown cost savings associated with HIT because it facilitates ordering of laboratory tests, use of bar codes to reduce errors in dispensing of medications in hospitals and distribution, and archiving of picture images [51–53]. This initiative has demonstrated that HIT that focuses on facilitating linkage and retention in HIV care, quality of HIV care, and adherence to ART may also reduce healthcare costs. Four of the 6 interventions we evaluated—including use of HIV surveillance data to identify out-of-care individuals, use of electronic laboratory ordering and prescribing, and development of a patient portal—were not only cost-effective but also cost saving.

In contrast to what we expected, costs associated with these HIT interventions were driven primarily by personnel time and not by costs associated with the purchase or maintenance of equipment and technology. In addition, over 3 years, personnel costs were evenly distributed across conceptualization and design of the HIT interventions, ongoing system maintenance and improvement, and user training and support. Interventions did not have defined start-up periods, but instead conducted iterative improvements over time. These findings correspond to previous research which found that ongoing yearly costs of HIT implementation were approximately 20% of initial costs [54].

We observed an increase in QALYs and a decrease in expected healthcare costs among patients in 4 of the 6 demonstration sites and a decrease in QALYs and an increase in healthcare costs among patients in 2 demonstration sites. These findings are primarily due to decreases in the proportion of patients with 0 to 200 cells/mm^3^ CD4 counts within Sites 3, 4, 5, and 6 because patients in this subgroup have substantially higher healthcare costs and higher mortality than other subgroups. Although we also observed significant increases in the proportion of patients on ART in Sites 3, 4, and 5, and significant increases in the proportion of patients with undetectable viral load in Sites 1, 3, 4, 5, and 6 [34], changes in the distribution of these characteristics had substantially less impact on short-term healthcare costs or the probability of mortality among patients with higher CD4 cell counts [40].

The 4 cost-saving interventions focused on linkage to HIV care (Site 3), improving access to HIV medications (Site 6), improving the quality of HIV care (Site 5), and improving patient access to information about their medical care (Site 4). The 2 interventions that were not cost-effective (Site 1 and Site 2) focused on expanding access to medical record information to support service providers. It is possible that these 2 interventions were less successful than other interventions because they did not result in targeted changes in staff workflow or behavior. In fact, it may well be that the current availability of user interfaces is not adequate to optimize or improve the provider experience at all. As Dr. Robert Wachter has pointed out, “[o]ur iPhones and their digital brethren have made computerization look easy, which makes our experience with health care technology doubly disappointing. An important step is admitting that there is a problem, toning down the hype, and welcoming thoughtful criticism, rather than branding critics as Luddites.” [55].

These results are relatively insensitive to variation in the parameters used to estimate cost-effectiveness. Under 2 scenarios, the Site 2 intervention was no longer cost saving. Furthermore, the additional cost per QALY gained under these scenarios (i.e., $3,087,353 and $39,397,160) dramatically exceeds baseline costs per QALY. This intervention was susceptible to variation in parameters because the distribution in health states was similar before and after implementation of the intervention (i.e., it contributed to a small number of QALYs gained).

These results must be interpreted within a limited context. First, because this initiative consisted of 6 demonstration projects implemented at the health facility or system level, we are not able to isolate the effect of health IT interventions implemented as part of this initiative from other existing interventions at the sites or from temporal trends. We cannot rule out the possibility that interventions outside of the current SPNS initiative contributed to changes in the prescription of ART, the proportion of patients with 0 to 200 cells/mm^3^ CD4 counts, or the proportion of patients with undetectable viral load. However, provider workflows and procedures in the 4 successful interventions changed substantially as a result of these interventions [36]. Therefore, we have reason to believe that the interventions contributed to meaningful changes in clinical practices and other outcomes. Second, for 3 of the 6 interventions, the population of people affected by the intervention changed over time. Site 1 included “high need” patients who exited the program once they were able to achieve undetectable viral load. Therefore, it is possible that this intervention benefited patients; however, we were not able to observe these patients over an extended period of time and thus were not able to observe substantial changes in CD4 cell count, the factor most associated with the cost of care over time. Of note, this intervention was maintained by Site 1 after completion of the initiative. Similarly, Sites 3 and 4 experienced substantial increases in the number of patients in the intervention over the life of the initiative. Although we have adjusted for differences in patient characteristics over time, it is possible that patients who were new to the site differed from existing patients and these differences explain the observed results. Third, because the care and treatment of people with HIV is complex, we are not able to identify the specific mechanisms through which these HIT interventions effect prescription of ART, the proportion of patients with 0 to 200 cells/mm^3^ CD4 counts or the proportion of patient with undetectable viral load. However, we are able to hypothesize how each intervention improves the quality of care for HIV–infected patients in each participating site. Fourth, this study was conducted some time ago. In the intervening years, many interventions that employ HIT have been shown to be cost-effective, including those that reduce the time needed for care, reduce duplication of care, and reduce unnecessary visits. However, to date, no studies have been conducted on the cost-effectiveness of the use of HIT in the context of HIV or other chronic diseases [56].

These findings contribute to a growing literature on the benefits of HIT and counter beliefs that the costs associated with HIT outweigh the potential benefits [53,56–63]. Previous reviews have demonstrated improvements in efficiency and reduced transcription errors. However, data on the effect of HIT on the costs of care and patient outcomes have been limited [59,62–64]. Many authors have suggested that HIT may only be cost effective in large health systems, but other evidence suggests that it may also be cost saving in smaller settings [55,59]. Other findings suggest that the scale of benefits and cost savings may depend on the “fit” of the HIT to the setting, how and how fully HIT is adopted and utilized, and whether HIT is coupled with behavior changes [59,61–64]. Our findings reinforce the role of these factors in that interventions that facilitated targeted changes in provider work flows were found to be cost saving, whereas interventions that facilitated access to information for patients or support service providers were less likely to be effective, cost-effective, or cost saving. The findings presented here suggest that HIT investments may contribute to improvements in effective treatment of HIV to achieve sustained viral suppression, but also may reduce the cost of care for PLHIV.

## Supporting information

S1 CHEERS Checklist (DOCX)

S1 Evaluation Protocol (DOCX)

S1 Text (DOCX)

## Abbreviations:

ART: antiretroviral therapy
EHR: electronic health record
HIT: health information technology
HRSA/SPNS: Health Resources and Services Administration’s Special Projects of National Significance
NHAS: US National HIV/AIDS Strategy
PLHIV: people living with HIV
QALY: quality-adjusted life-year
RWHAP: Ryan White HIV/AIDS Program
SPNS: Special Projects of National Significance
UCSF: University of California, San Francisco
