# Supplementary material for: Health information technology interventions and engagement in HIV care and achievement of viral suppression in publicly funded settings in the US: A cost-effectiveness analysis
Source: PLoS Med. 2021 Apr 7;18(4):e1003389. doi: 10.1371/journal.pmed.1003389 (PMC8059802; doi:10.1371/journal.pmed.1003389)
Supplement: S1 Evaluation Protocol — (DOCX) [file pmed.1003389.s002.docx]

**Health Information Technology Evaluation and Support Center**

**Multisite Evaluation Protocol**

1. **Synopsis**

The Health Information Technology Evaluation and Support Center (HITEC) is a center to support demonstration projects implementing and evaluating electronic network systems as part of the Special Projects of National Significance (SPNS) Initiative on Information Technology Networks of Care. Electronic network systems are comprised of computer hardware and software programs that permit the electronic exchange of health information among providers at different points of service, thereby improving the continuity and quality of care. HITEC will provide leadership in the design, implementation, and evaluation of such electronic network systems among providers serving people living with or affected by HIV/AIDS in underserved communities. This will be accomplished using both qualitative and quantitative methods. Qualitative, key informant interviews will help characterize the clinical settings, providers, and patients at the demonstrations sites funded under this Initiative; and contextualize the barriers, facilitators, and circumstances that affect the implementation of electronic network systems at these sites. Quantitative patient and provider surveys, as well medical records reviews, will be used to collect common indicator variables at all sites in order to measure the implementation of the network systems and their impact on patient care. The primary goal of these mixed methods is ensure that the evaluation of electronic network systems has maximum impact on the theory, practice, and policy of delivering HIV health services. In addition, HITEC will provide training and support to sites implementing and evaluating electronic networks. This will be accomplished through site visits, grantee meetings, in-person trainings, and ongoing technical assistance. The work of HITEC will be conducted by a multidisciplinary team led by the Center for AIDS Prevention Studies (CAPS) at the University of California, San Francisco (UCSF).

1. **Hypothesis**

As an evaluation center, this work will be guided by a set of evaluation questions, as opposed to scientific hypotheses. These questions are:

1. What are major barriers—technological, attitudinal, financial, and structural—to the implementation of electronic network systems in Ryan White Program-funded clinics?
2. What characteristics of electronic network system technology are associated with improved decision-making and greater satisfaction among clinical providers?
3. What characteristics of electronic network systems and the clinics in which they are used are associated with cost-effective improvements in patient treatment outcomes, such as linking to care at an earlier stage in HIV disease, better tracking of HIV disease progression indicators, and better tracking of referrals to care?
4. What are the characteristics of patient populations that are more receptive to the use of health information technology in clinical settings?

How do electronic network systems enhance linkages between local clinics and public health architectures, such as facilitating the completion of Ryan White Program reports?

1. **Specific Aims**

- Aim 1 (Evaluation): To facilitate and conduct rigorous evaluation across demonstration sites that has maximum impact on the practice and policy of using electronic network systems in settings that provide care and support for people living with or affected by HIV.
- Aim 2 (Support and Training): To provide methodological (both quantitative and qualitative) research design and evaluation consultation and support to proposed demonstration projects; to offer technical assistance on the implementation of electronic network systems in HIV care settings; to assist sites in the design of state-of-the-art data collection and management systems; and to provide a central database for the measurement of outcomes.
- Aim 3 (Dissemination): To synthesize and disseminate findings from demonstration projects so that they have optimum impact on the research, practices, and policies of electronic network systems.
- Aim 4 (Capacity): To stimulate innovative projects and ensure scientific excellence, as well as support organizational capacity to ensure integrity of research and sound fiscal operations.

1. **Background and Significance**

Interest has grown in the application of computer technology to health information, including the use of electronic network systems, because research has documented the potential for success. A recent review of published articles on computer technology in primary care settings found evidence that it can improve the management of chronic diseases, increase the rate with which preventive medical tasks are completed (e.g., vaccinations), increase the prescription of generic drugs, and promote cost savings and the administration of fewer unnecessary tests.^9^ More recent research has continued documenting these successes by showing that computer technology leads to better recording of the clinical indicators of chronic conditions,^10^ better patient outcomes for hypertension,^11, 12^ fewer red blood cell transfusions and associated costs,^13^ and an increase in the quality of nursing by promoting more direct interaction time with patients.^14^

Electronic network systems have the ability to connect health information with providers, patients, and public health agencies, which could be particularly valuable in the area of HIV care. By its very nature, HIV disease is complex, affecting patients’ health and well-being in multiple ways. As such, promoting the communication of information among various specialized providers could enhance greatly patient care in a number of domains:

**Diagnosis.** The advantages of electronic technology may start as early as the identification of disease. Research has documented systematic disparities in the diagnosis of HIV disease, with ethnic minorities and women receiving their diagnoses later than Whites and men.^15-19^ When examined retrospectively, research has found that many patients pass through the medical care system with evidence of HIV transmission risks and yet are never screened.^20-22^ This phenomenon occurs in part because seroconversion symptoms are often misdiagnosed ^23-26^ or because such symptoms are absent altogether.^26^ However, recent research has demonstrated that it is possible to improve the likelihood of identifying patients with very recent infections. By collecting information on specific clinical features commonly associated with HIV seroconversion, patients at highest risk of having new infections can be accurately identified and offered HIV testing.^27^ Electronic network systems could aid this goal by allowing providers to record clinical and behavioral information related to possible HIV seroconversion, and then by generating electronic reminders for testing when patients are at high risk of infection.

**Treatment.** Electronic technology will likely be beneficial in the ongoing treatment of the disease. For many of the most vulnerable communities affected by HIV, referrals to care are a critical challenge. For example, research with homeless and low-income populations has shown that structural and individual barriers often prohibit access to services even when care is desired.^28-33^ To achieve improvements in health in these marginalized populations, one needs active outreach efforts and coordinated care.^34-37^ Electronic network systems would allow for improved continuity of care and referrals by enabling one to transmit needed medical information directly and instantly, without having to rely on courier services or the patient to carry it; store critical tracking information to help locate difficult-to-reach patients; and allow one to determine whether a patient actually acts on a referral. Electronic network systems also would enable providers to determine when critical tests were conducted last, to access the results of those tests regardless of whether or not they occurred in the office where the provider works, and to ensure that further care is delivered in a timely manner. For example, in prior research, electronically-generated reminders based on patient records reduced significantly the amount of time that elapsed before a provider ordered a needed CD4 test or prescribed antiretroviral medications to individuals with declining immune function.^38^

**Preventing Transmission.** Increasing emphasis is being placed on clinic-based efforts to work with patients to limit the likelihood of disease transmission.^39, 40^ These “Prevention with Positives” initiatives face a number of challenges, including a lack of relevant provider training,^3, 41^ insufficient time,^3, 42^ a belief that prevention efforts will fail,^5^ and systematic disparities in the delivery of such care.^4, 41-43^ However, other research has shown that relatively brief, patient-tailored, clinician-delivered prevention messages can significantly reduce sexual risk behaviors.^44^ Electronic network systems can collect relevant behavioral risk data from multiple sources to help clinicians tailor risk reduction messages. Furthermore, electronic network systems can contribute to partner notification efforts by transmitting patient-volunteered partner information to individuals who will work with the patient on disclosing his or her infection.

**Challenges to Implementation**

The application of electronic technology to health information is still relatively new and faces barriers to wide adoption. Little research has focused specifically on barriers to electronic network systems, but investigators have examined the obstacles to computer technology more generally.^6^ In particular, there are four important barriers that likely impact the acceptability and adoption of electronic network systems:

1. Cost (both in terms of finances and personnel). Electronic network systems require an initial investment in hardware, networking, and provider and staff training. There are reports of primary care training centers discontinuing the use of computer-based systems because the investments required were too burdensome.^45^
2. Data security. The Health Insurance Portability and Accountability Act (HIPAA) of 1996 has strict standards to protect patient information, and limits to whom such information can be disclosed. An electronic network system must have sufficient data encryption and firewall protections to ensure that patient information is not compromised. Furthermore, users of the system must ensure that the protocols for sharing and accessing patient information within the network are in compliance with HIPAA regulations.
3. Attitudes toward electronic network systems. Many providers have concerns about the time and training that are required to learn to use new programs.^9, 46^ Patients have voiced concerns about the security of the data in computer systems,^47, 48^ and even indicated an unwillingness to see providers whom they knew were entering medical information into such systems.^49^ Privacy concerns are especially important around HIV, which is often still stigmatized.^50^
4. Program compatibility.^6, 51^ To be useful, electronic data collected in one computer application must be readable by the other applications in use in different provider offices. If compatibility is lacking, use of the system or favorable opinions about it may lag.

*Summary*

Electronic network systems are a promising and important component of health information technology. Such systems could improve diagnosis, treatment, and prevention efforts with HIV-infected clients, and may enhance policy development at a national level. However, there remain significant barriers to the adoption of these systems, which could undermine the uptake and use of electronic technology. A thorough evaluation would help ensure that electronic systems are implemented in a manner that maximizes the likelihood of success.

1. **Design**

HITEC’s evaluation consists of five key components displayed below. The HRSA-sponsored Initiative (for which HITEC is the evaluation center) is nearing the end of Year 2. Assessments described as occurring “Before ENS Rollout” are nearly completed. After ENS rollout, HITEC is continuing to collect follow-up data in six month intervals for two years, until approximately September, 2010. The final year of the Initiative (Sept. 2010- Aug. 2011) will be dedicated to data analysis and dissemination.

| **Method** | **Population** | **Sample size/**  **Demonstration Site** | **Time frame** |
| --- | --- | --- | --- |
| **Key Informant interviews (Component 1)** | Project Staff and Key Collaborators | Up to 7 | Before ENS rollout and late in Year 3 of Initiative |
|  | Users of the ENS | Up to 10 | Before ENS rollout and late in Year 3 Initiative |
|  | Patients | Up to 10 | In Year 3 of Initiative |
| **Surveys**  **(Component 2-4_** | Systems survey  (Component 2) | 1 survey completed for each site per wave (completed via combined interview with project director and network administrator) | Before ENS rollout, every 6 months after rollout, at the end of Initiative |
|  | Users Survey (Component 3) | Entire population (about 20 at most sites) | Before ENS rollout and every 6 months after rollout |
|  | Patients Survey  (Component 4) | 100 | Before ENS rollout and every 6 months after rollout |
| **ENS data abstraction (Component 5)** | Patients | 100 | Before ENS rollout and every 6 months after rollout |

Key informant interview capture data that speak to the facilitators and barriers to implementation of the ENS. The systems, users, and patients survey, as well as the ENS data abstraction, capture quantitative data that allow HITEC to examine the system, user, and patient characteristics associated with the greatest improvements in clinical care and health outcomes.

1. **Statistical Analysis**

**Key Informant Interviews:** Qualitative data will be evaluated by our team of research analysts, using a method for codebook development and validation that has been developed over years of work with similar projects and populations. The procedures we use are as follows: First, all interviews are tape-recorded and transcribed. If an interview is conducted in Spanish, it is translated into English before transcription. Second, analysts read a cross-section of the interview transcripts and establish a preliminary codebook. Third, the preliminary codebook is applied to a sub-set of interviews. As each analyst attempts this process, he or she modifies the codes to reflect further nuances in conceptual categories. After preliminary coding is complete, the analysts meet and settle on a final set of codes. Fourth, every interview transcript is read independently by two analysts who apply the final codes to the text. The analysts then meet to compare their codes. Disagreements are resolved by discussion to reach consensus. *Atlas.ti*^52^ is used to facilitate all coding work.

Once coding is finished, analysts then examine the complete set of interviews to identify convergent and divergent themes that have emerged across various conceptual categories. For example, analysts might examine the similarities and differences in facilitators and barriers to electronic network systems across demonstration sites, types of providers, or target populations.

**Quantitative Data Analyses (includes data collected via the systems survey, user survey, patient survey, and ENS data extraction):**

***Analyses will be organized around a series of objectives:***

**Quantitative Objective 1. *To assess whether Electronic Network Systems (ENS) improve the quality of care, satisfaction with care, and patient health outcomes.***

We will present descriptive analyses of the quality of HIV care, support services, and patient health outcomes over the project period (graphs, etc.). We also will assess changes in the quality of HIV care, support services, and patient outcomes using both descriptive analyses and mixed models to adjust for baseline differences in each outcome across the six ENS and to account for the covariance of outcomes within each ENS.

**Quantitative Objective 2.  *To assess what characteristics of the ENS are specifically associated with improvements in care, satisfaction, and health outcomes.***

Data collected as part of the “systems” assessment will allow us to characterize the components, functions, and data of the six ENS systems. We will present descriptive analyses that demonstrate the relationship between specific ENS characteristics and outcomes in quality of care, patient satisfaction, and health status. Whenever possible, we will categorize the six ENS into subgroups based on common characteristics and examine via descriptive analyses the associations of the subgroups with clinical outcomes.

**Quantitative Objective 3.** ***To assess how user and patient characteristics and attitudes mediate and/or moderate changes in outcomes.***

We specifically expect that ENS, user, and patient characteristics will influence users’ attitudes about the system. These attitudes in turn will affect actual system usage, which will affect health care service delivery and, ultimately, health outcomes. Relationships between individual variables can be documented via correlations or via regression equations. Potential mediation can be examined by regressing outcomes variables (e.g., health care service delivery) onto predictor variables (e.g., user attitudes) in models that include and exclude potential mediators (e.g., actual system usage). If the mediator is in fact responsible for the relationship between the predictor and the outcome, then the regression weight for the predictor will be reduced significantly whenever the mediator is included in the regression model. Potential moderation can be examined by including interaction terms in regression models. If we have sufficient power, the overall quality of the conceptual model can be tested using structural equation modeling.

**Quantitative Objective 4. *To assess the cost-effectiveness of each ENS.***

The final objective uses data collected as part of the systems assessment. We will be capturing information on the total investment each site must make in its ENS, both in terms of financial commitments and in terms of personnel hours. This information will allow us to describe the total amount of money and effort expended in relationship to the degree of changes in services delivered, patient satisfaction, and HIV-related health outcomes. To compare these values across sites, one can standardize and combine cost variables (money + effort), then standardize outcome variables (e.g., health outcomes) and compute a ratio of <costs/outcome> for each of the six ENS. Because the ratios are standardized, one is able to compare the relative success of the six ENS at promoting desired outcome changes at lower costs. Cost effective systems are those that achieve relatively greater (desired) changes for relatively less money or personnel hours expended.

1. **Sample Size**

The table below helps display how many participants are being collected at each site per wave.

| **Method** | **Population** | **Sample size/**  **Demonstration Site/Wave of Assessment** | **Number of Assessments** |
| --- | --- | --- | --- |
| **Key Informant interviews (Component 1)** | Project Staff and Key Collaborators | Up to 7 | Two assessments: Before ENS rollout and in Year 3 of Initiative |
|  | Users of the ENS | Up to 10 | Two assessments: Before ENS rollout and in Year 3 Initiative |
|  | Patients | Up to 10 | One assessment: In Year 3 of Initiative |
| **Surveys**  **(Component 2-4)** | Systems survey  (Component 2) | 1 survey completed for each site per wave (completed via combined interview with project director and network administrator) | Six assessments: Before ENS rollout; every 6 months after rollout (total of 4 assessments); at the end of Initiative |
|  | Users Survey (Component 3) | Entire population (about 20 per site) | Five assessments: Before ENS rollout and every 6 months after rollout (total of 4 assessments post rollout) |
|  | Patients Survey  (Component 4) | 100 | Five assessments: Before ENS rollout and every 6 months after rollout (total of 4 assessments post rollout) |
| **ENS data abstraction (Component 5)** | Patients | 100 | Five assessments: Before ENS rollout and every 6 months after rollout (total of 4 assessments post rollout) |

For **Key Informant Interviews**, we may interview up to 17 project staff and ENS users per site before ENS rollout and again in Year 3 of the HRSA-sponsored Initiative. It is likely, but not guaranteed, that many of these individuals will be the same across the two waves. In the event that all interviews are with different individuals, then we will speak with a total of 204 individuals (17 per site per wave; 6 sites; 2 waves). We also will interview 10 patients per site in Year 2 of the Initiative (total: 60). **Thus, the total maximum number of unique individuals that may be interviewed is up to 264.** We based the projected number of interviews on our prior work as an evaluation center of HIV prevention services in clinical settings. The 264 key information interview participants will be enrolled by UCSF.

For the **systems survey**, we will complete one survey per site per wave (total of six waves of assessments). To complete these surveys, we must interview project personnel with knowledge of both the ENS and of the project expenses. As such, we will likely talk to both the local project director and the local network administrator at each site for each wave of data collection. (Note: the people interviewed at each site at each wave will complete the survey together. In other words, we will interview two people per site per wave in order to obtain one completed survey per site per wave.) The participants will likely be the same across waves. In the event that the interviewed individuals are different at each wave, then will speak with **up to 72 unique individuals** (2 people site per wave; six sites; six waves). The systems survey is meant to capture accurate the current configuration of the system. We are opting to include in the interviews those people most likely to have relevant knowledge about the system. The 72 people interviewed for the systems survey will be enrolled as participants by UCSF.

For the **user survey**, we will seek to interview the entire population of users at the sites. The exact number of users differs by site, but is an average of about 20. The same users will likely be assessed across waves. However, if all users are unique at each wave, then the total number of people interviewed will be **up to 600 unique individuals** (20 people per site per wave; six sites; five waves). Because the total population of users at each site is relatively small, we are seeking to survey them all in order to maximize statistical power for analyses. Users will enrolled as participants at UCSF.

For the **patient survey**, we will seek to interview 100 individuals per wave. If all individuals are unique at each wave, then we may survey **up to 3000 unique individuals** (100 people per site per wave; 6 sites; 5 waves). A sample of 100 per site (N=600 per observation period) will give us 80% power to detect 10% change in proportional outcomes (e.g. 50% to 60%) in the underlying popluation using a two-sided test with 0.05 alpha. This estimate assumes an intra-class correlation (ICC) of 0.005. ***Participants in this survey (except pilot test subjects) will be enrolled at the local sites via procedures approved by the local IRB. UCSF will only receive de-identified data for secondary analyses.***

For the **ENS data extraction**, we will seek to obtain de-identified data on 100 individuals per wave. (Note: these individuals do NOT have to be the same people who participate in the patient survey.) If all individuals are unique at each wave, then we may obtain data on **up to 3000 people** (100 people per site per wave; 6 sites; 5 waves). A sample of 100 per site (N=600 per observation period) will give us 80% power to detect 10% change in proportional outcomes (e.g. 50% to 60%) in the underlying popluation using a two-sided test with 0.05 alpha. This estimate assumes an intra-class correlation (ICC) of 0.005.. ***Data extraction is conducted at the local sites via procedures approved by the local IRB. UCSF will only receive de-identified data for secondary analyses.***

**If every participant were unique (across all components), then up to 6936 individuals could contribute data to the study.**
