# Supplementary material for: Health information technology interventions and engagement in HIV care and achievement of viral suppression in publicly funded settings in the US: A cost-effectiveness analysis
Source: PLoS Med. 2021 Apr 7;18(4):e1003389. doi: 10.1371/journal.pmed.1003389 (PMC8059802; doi:10.1371/journal.pmed.1003389)
Supplement: S1 Text — (DOCX) [file pmed.1003389.s003.docx]

**Table A: Model Inputs – Distribution of disease states at baseline and follow-up by site**

|  | **Site 1** | **Site 2** | **Site 3** | **Site 4** | **Site 5** | **Site 6** |
| --- | --- | --- | --- | --- | --- | --- |
| **Distribution of disease states at baseline** | | | | | | |
| % on ART, CD4 > 500, viral load > 1000 | 0.0 | 0.6 | 0.0 | 7.3 | 1.8 | 1.0 |
| % on ART, CD4 > 500, viral load < 1000 | 30.6 | 26.4 | 13.6 | 33.3 | 29.3 | 33.9 |
| % on ART, CD4 201-500, viral load > 1000 | 4.2 | 5.0 | 9.1 | 17.4 | 10.1 | 4.2 |
| % on ART, CD4 201-500, viral load < 1000 | 40.4 | 37.9 | 22.7 | 15.9 | 26.6 | 34.9 |
| % on ART, CD4 < 200, viral load > 1000 | 4.2 | 6.6 | 15.9 | 10.1 | 6.4 | 3.0 |
| % on ART, CD4 < 200, viral load < 1000 | 12.5 | 6.6 | 13.6 | 7.3 | 12.8 | 7.2 |
| % not on ART, CD4 > 500, viral load > 1000 | 0.0 | 6.6 | 4.6 | 0.0 | 2.8 | 2.7 |
| % not on ART, CD4 > 500, viral load < 1000 | 5.6 | 2.2 | 6.8 | 2.9 | 3.7 | 3.0 |
| % not on ART, CD4 201-500, viral load > 1000 | 1.4 | 3.3 | 4.6 | 4.4 | 1.8 | 5.0 |
| % not on ART, CD4 201-500, viral load < 1000 | 1.4 | 3.3 | 6.8 | 1.5 | 3.7 | 3.0 |
| % not on ART, CD4 < 200, viral load > 1000 | 0.0 | 0.0 | 0.0 | 0.0 | 1.0 | 1.5 |
| % not on ART, CD4 < 200, viral load < 1000 | 0.0 | 1.7 | 2.3 | 0.0 | 0.0 | 0.5 |
| **Distribution of disease states at follow-up** | | | | | | |
| % on ART, CD4 > 500, viral load > 1000 | 1.3 | 0.6 | 2.1 | 8.8 | 4.5 | 2.6 |
| % on ART, CD4 > 500, viral load < 1000 | 21.9 | 40.8 | 6.3 | 38.2 | 43.0 | 49.9 |
| % on ART, CD4 201-500, viral load > 1000 | 10.5 | 3.9 | 10.4 | 5.9 | 3.0 | 4.7 |
| % on ART, CD4 201-500, viral load < 1000 | 26.5 | 28.5 | 18.8 | 29.4 | 27.5 | 33.3 |
| % on ART, CD4 < 200, viral load > 1000 | 16.0 | 6.7 | 12.5 | 2.9 | 6.0 | 3.0 |
| % on ART, CD4 < 200, viral load < 1000 | 12.4 | 10.6 | 12.5 | 5.9 | 4.5 | 4.7 |
| % not on ART, CD4 > 500, viral load > 1000 | 3.6 | 1.7 | 6.3 | 0.0 | 4.0 | 0.5 |
| % not on ART, CD4 > 500, viral load < 1000 | 2.6 | 2.2 | 4.2 | 5.9 | 4.0 | 0.4 |
| % not on ART, CD4 201-500, viral load > 1000 | 2.9 | 2.2 | 18.8 | 0.0 | 0.5 | 0.5 |
| % not on ART, CD4 201-500, viral load < 1000 | 0.6 | 2.2 | 6.3 | 2.9 | 2.5 | 0.0 |
| % not on ART, CD4 < 200, viral load > 1000 | 1.3 | 0.6 | 2.1 | 0.0 | 0.5 | 0.5 |
| % not on ART, CD4 < 200, viral load < 1000 | 0.3 | 0.0 | 0.0 | 0.0 | 0.0 | 0.0 |

**Table B: Model Inputs – Probability of HIV-related mortality (per month)**

| **Variable** | **Value** | **Range** | **References** |
| --- | --- | --- | --- |
| ***Patients without AIDS*** |  |  |  |
| CD4 < 200, viral load <1000 | 0.0048 | (0.0024, 0.0096) | [39] |
| CD4 < 200, viral load > 1000 | 0.0053 | (0.0027, 0.0106) | [39] |
| CD4 201-500, viral load < 1000 | 0.0011 | (0.0006, 0.0022) | [39] |
| CD4 201-500, viral load > 1000 | 0.0019 | (0.0010, 0.0038) | [39] |
| CD4 > 500, viral load < 1000 | 0.0006 | (0.0003, 0.0012) | [39] |
| CD4 > 500, viral load > 1000 | 0.0010 | (0.0005, 0.0020) | [39] |
| ***Patients with AIDS*** |  |  |  |
| CD4 < 200 | 0.0117 | (0.0059, 0.0234) | [39] |
| CD4 201-500 | 0.0020 | (0.0010, 0.0040) | [39] |
| CD4 > 500 | 0.0006 | (0.0030, 0.0120) | [39] |

**Table C: Model Inputs – Transition Probabilities (per month) [39]**

| **Not on ART** |  |  |  |  |  |  |
| --- | --- | --- | --- | --- | --- | --- |
|  | cd4 0-200, vl >= 1000 | cd4 201-500, vl >= 1000 | cd4 > 500, vl >= 1000 | cd4 0-200, vl <1000 | cd4 201-500, vl < 1000 | cd4 > 500, vl < 1000 |
| cd4 0-200,  vl >= 1000 | 1 | 0 | 0 | 0 | 0 | 0 |
| cd4 201-500, vl >= 1000 | 0.0004 | 0.9996 | 0 | 0 | 0 | 0 |
| cd4 > 500,  vl >= 1000 | 0 | 0.0027 | 0.9973 | 0 | 0 | 0 |
| cd4 0-200,  vl <1000 | 0 | 0 | 0 | 0.953 | 0.047 | 0 |
| cd4 201-500, vl < 1000 | 0 | 0 | 0 | 0 | 0.9656 | 0.0344 |
| cd4 > 500,  vl < 1000 | 0 | 0 | 0 | 0 | 0 | 1 |
| **On ART** |  |  |  |  |  |  |
|  | cd4 0-200, vl >= 1000 | cd4 201-500, vl >= 1000 | cd4 > 500, vl >= 1000 | cd4 0-200, vl <1000 | cd4 201-500, vl < 1000 | cd4 > 500, vl < 1000 |
| cd4 0-200,  vl >= 1000 | 0.89469 | 0.00531 | 0 | 0.09941 | 0.00059 | 0 |
| cd4 201-500, vl >= 1000 | 0 | 0.89379 | 0.00621 | 0 | 0.09931 | 0.00069 |
| cd4 > 500,  vl >= 1000 | 0 | 0 | 0.9 | 0 | 0 | 0.1 |
| cd4 0-200,  vl <1000 | 0 | 0 | 0 | 0.941 | 0.059 | 0 |
| cd4 201-500, vl < 1000 | 0 | 0 | 0 | 0 | 0.9576 | 0.0424 |
| cd4 > 500,  vl < 1000 | 0 | 0 | 0 | 0 | 0 | 1 |

**Table D: Model Inputs – Utilities (quality-adjusted life year for each disease state)**

| **Variable** | **Value** | **Range** | **References** |
| --- | --- | --- | --- |
| CD4 <=200, viral load >= 1000 | 0.8150 | (0.6300, 0.9075) | [40] |
| CD4 201-500, viral load >= 1000 | 0.9317 | (0.8534, 0.9659) | [40] |
| CD4 > 500, viral load >= 1000 | 0.9380 | (0.8760, 0.9690) | [40] |
| CD4 <=200, viral load < 1000 | 0.8220 | (6.4400. 0.9110) | [40] |
| CD4 201-500, viral load < 1000 | 0.9315 | (0.8630, 0.9658) | [40] |
| CD4 > 500, viral load < 1000 | 0.9540 | (0.9080, 0.9770) | [40] |

**Table E: Model Inputs – Healthcare Costs**

| **Costs of care (2019 US dollars)** | | | |
| --- | --- | --- | --- |
| **Annual HIV-related healthcare costs** | | | |
| ***Patients on ART*** |  |  |  |
| CD4 < 200, viral load <1000 | $11,840 | 8,880 – 17,760 | [39-46] |
| CD4 < 200, viral load > 1000 | $9,709 | 7,282 – 14,564 | [39-46] |
| CD4 201-500, viral load < 1000 | $7,356 | 5,517 – 11,034 | [39-46] |
| CD4 201-500, viral load > 1000 | $6,620 | 4,965 – 9,938 | [39-46] |
| CD4 > 500, viral load < 1000 | $4,915 | 3,686 – 7,373 | [39-46] |
| CD4 > 500, viral load > 1000 | $4,325 | 3,244 – 6,485 | [39-46] |
| ***Patients not on ART*** |  |  |  |
| CD4 < 200, viral load <1000 | $26,018 | 19,514 – 39,027 | [39-46] |
| CD4 < 200, viral load > 1000 | $21,225 | 15,919 – 31,838 | [39-46] |
| CD4 201-500, viral load < 1000 | $8,251 | 6,188 – 12,377 | [39-46] |
| CD4 201-500, viral load > 1000 | $7,426 | 5,570 – 11,139 | [39-46] |
| CD4 > 500, viral load < 1000 | $4,915 | 3,686 – 7,373 | [39-46] |
| CD4 > 500, viral load > 1000 | $4,325 | 3,244 – 6,488 | [39-46] |
| **Cost of antiretroviral therapy** | $25,049 | 14,729-38,165 | [41, 47-50] |
| **Cost of non HIV-related health care** | $9,026 | 6,770 – 13,539 | [41] |
